# Fungus-Growing Ants: Models for the Integrative Analysis of Cognition and Brain Evolution

**DOI:** 10.3389/fnbeh.2020.599234

**Published:** 2020-12-11

**Authors:** Isabella B. Muratore, James F. A. Traniello

**Affiliations:** ^1^Department of Biology, Boston University, Boston, MA, United States; ^2^Graduate Program in Neuroscience, Boston University, Boston, MA, United States

**Keywords:** cognitive ecology, behavior, social brain, ant, division of labor

Agents of selection for behavioral responses to abiotic, biotic, and social environments are described as *cognitive challenges*. Research integrating behavior, ecology, and brain evolution has generated a growing literature—and sometimes controversy—over inferences made from correlating cognitive traits with neural metrics. We propose that our understanding of the role of cognition in brain evolution can be advanced through studies of eusocial insect species differing in agricultural practices and degree of division of labor, and thus social complexity. Fungus-growing ants offer diverse systems to assess the impacts of cognitive challenges on behavioral evolution and its neural and genomic architectures. Workers exhibit variability in social role differentiation in association with diet, morphology, group size, and task efficiency. This suite of covarying traits enables the accurate mapping of cognition, worker repertoire breadth, neuroanatomy, and genomic change in light of social evolution.

## How Do Brains Respond to Cognitive Challenges?

Cognition is difficult to universally define (Logan et al., 2018; Bayne et al., 2019) and measure (Rowe and Healy, 2014; Simons and Tibbetts, 2019). However, cognitive ecologists have developed definitions emphasizing divergent demands from behavioral niches and neurobiological capabilities (Balda and Kamil, 1989; Real, 1993; Shettleworth, 2000, 2010; Dukas and Ratcliffe, 2009; Lihoreau et al., 2019). Cognition should be linked to ecological adaptation to understand developmental and evolutionary brain plasticity. Cognitive capability is thus the product of selection for brain organization to adaptively increase computational power and reduce energetic costs. Metrics applied in the study of brain evolution range from genes and cells to nervous system topologies. Correlations between behavioral capabilities and tissue volume have been viewed critically (Herculano-Houzel et al., 2006, 2007; Healy and Rowe, 2007, 2013; Chittka and Niven, 2009; Godfrey and Gronenberg, 2019; Wartel et al., 2019), although in principle quantify brain investment. Functionally specialized brain compartments may develop allometrically (disproportionate scaling) through differential cell and tissue-type trajectories (Barton and Harvey, 2000; Hager et al., 2012), circuitry (Guzowski et al., 2005), neuron structure and function (Quiroga et al., 2005), and genetics (Hibar et al., 2015; Kohno and Kubo, 2019). These patterns provide fine-grain traits for evolutionary analyses.

Social environments can influence brain evolution. Primates distinguish rivals from allies and recall interaction histories. Social brain theory, which posits a positive correlation between brain volume and group size to track social relationships (Dunbar and Shultz, 2017), has been applied to eusocial insects (Lihoreau et al., 2012; Godfrey and Gronenberg, 2019). However, eusocial insect workers typically lack the competing demands of direct reproduction; their brains are functionally dedicated to altruistic labor, and cognitive challenges from specialized behavior can thus be more clearly circumscribed. Diverse social systems enable the functional analysis of mosaic brains and responsiveness to divergent sensory demands underpinning task specialization (Muscedere and Traniello, 2012; Giraldo et al., 2013; Gordon et al., 2017). Two eusocial insect clades—a tribe of ants and a subfamily of termites—include *ultrasocial* species (Campbell, 1982) that are agriculturalists, producing their own crops of gongylidia—nutritional fungal swellings—and have evolved complex division of labor. These traits are shared with humans (Gowdy and Krall, 2016). Ant societies, as models, can be experimentally dissected (Kennedy et al., 2017), enabling studies of cognitive variation in association with the evolution of division of labor.

Assessing motivated behavior in natural contexts (Rowe and Healy, 2014) and selecting comparative frameworks illustrating divergence in cognitive challenges across related species (Simons and Tibbetts, 2019) are essential to link fitness to behavioral evolution. Therefore, to determine cognitive impacts on brain evolution, a model system should meet the following criteria: (1) the natural behavioral environment can be measured to assess sensory and processing requirements; (2) behavior can be quantified at multiple levels of intraspecies and interspecies biological organization; and (3) the metrics used to identify neural and genomic underpinnings are methodologically and statistically robust. With these points in mind, we identify fungus-growing ants as appropriate and insightful study models for cognitive evolution.

## Division of Labor and Worker Cognition

The evolution of division of labor in support of agriculture in fungus-growing ants enables societal and individual cognition to be examined. Workers vary morphologically (monomorphism to exceptional polymorphism) and behaviorally (task pluripotency to specialization) across species and within colonies (Mehdiabadi and Schultz, 2010). In highly polymorphic leafcutting ants, colonies are large and may produce size-differentiated worker*s*—for example, minims, medias, and majors in order of increasing size. This variation in body size, colony size, and diet can help disentangle confounding factors that may obscure the linkage of neuroanatomy to behavior. Fungus-growing ants select, harvest, and process plant tissue and other substrates to provide for fungal growth, cultivate fungus, manage waste and control infection, construct and maintain the nest and regulate microclimate, and provide defense. Workers with specialized repertoires are predicted to be more efficient than generalists (Wilson, 1980b). In theory, drivers of worker task performance may differ, but in polymorphic species body size and behavior are integrated and clearly correlate (Beshers and Fewell, 2001). Cognitive needs vary according to role and worksite: tasks performed within the nest by fungal-garden tenders require different stimulus-processing capabilities than foragers or defenders working outside the nest or at multiple worksites. Identifying, cutting, transporting, and mulching leaves forms an assembly line of exterior to interior work where leaf fragments are degraded as they are passed from larger to smaller workers and eventually deposited as fungal mulch. Worker size-related labor therefore requires specific motivation and cognitive abilities.

Minim workers primarily transplant and prune gongylidia. Working in dark underground fungal chambers, they likely rely on sensory inputs other than vision for navigation, which may involve the central complex (Plath and Barron, 2015; Honkanen et al., 2019). They also nurse, recognizing larval needs and discriminating brood stages, and assess humidity and temperature to maintain optimal growth conditions. These tasks involve chemical signals (Schultner and Pulliainen, 2020) processed by the antennal lobes and mushroom bodies, as well as fine motor coordination of the mouthparts, mediated by subesophageal zone circuitry (Paul and Gronenberg, 2002). Minims may deposit pheromones on foraging trails (Howard, 2001; Evison et al., 2008), clean contaminants from incoming leaves and otherwise protect the fungus from microbes (Goes et al., 2020), and defend against parasitic flies (Feener and Moss, 1990).

Media workers engage in diverse tasks. Large-scale agriculture requires evaluating diverse plant chemistries to assess leaf quality and maximize fungal growth (Hubbell et al., 1984; Howard et al., 1988; Saverschek et al., 2010). This discrimination may require learning. Also, the gustatory and olfactory processing abilities of medias should be well developed. Media worker skill in cutting leaves (Wilson, 1980b) requires compass-like coordination of legs and mandibles that determines leaf fragment size, facilitating size-assortative load-bearing for transport (Wilson, 1980a; Burd, 2000; Burd and Howard, 2008). Medias navigate trails between food sources and the nest. In many ants, this process involves recalling landmarks, using odometry and optic flow to measure speed and distance, learning canopy patterns and celestial cues, and decoding chemical recruitment information (Ronacher and Wehner, 1995; Wittlinger et al., 2006; Provecho and Josens, 2009; Basten and Mallot, 2010; Müller and Wehner, 2010; Steck, 2012; Heinze et al., 2018). Media worker foraging thus requires processing multimodal signals through interplay between the antennal and optic lobes, mushroom bodies, and central complex. Behavioral flexibility may be reflected in enlarged mushroom bodies (Farris, 2013), a pattern expected in media brains, but not minims or majors.

Majors defend against army ants and other enemies (Powell and Clark, 2004). Defensive may require close-range vision, mediated by the optic lobes (Via, 1977), and antennal lobe and mushroom body tuning to recruitment and alarm pheromones (López-Riquelme et al., 2006; Mizunami et al., 2010). Differences in task biomechanical demands are evident in subcaste myology (Gronenberg et al., 1997; Paul and Gronenberg, 1999, 2002): larger mandibular muscles provide majors with bite force, controlled in part by the subesophageal zone.

## Social and Phylogenetic Perspectives on Cognitive Evolution

Fungus-growing ant species richness (>230 species; Schultz and Brady, 2008) encompasses exceptional heterogeneity in agricultural practice and social complexity. Behavioral phenotypes evolved greater specialization through developmental divergence in worker morphology (Mehdiabadi and Schultz, 2010; Sosa-Calvo et al., 2018; Solomon et al., 2019). The diversity of worker phenotypes in leafcutting genera such as *Atta* and *Acromyrmex*, which cultivate large quantities of fungus and form colonies of millions of polymorphic workers, is thought to have evolved from an ancestral monomorphic, generalist worker caste (Wilson, 1980a). The ancestral worker phenotype is evident in the paleoattini: these species form small colonies of monomorphic workers that engage in basic agriculture, scavenging insect frass and other materials for fungal substrate. Repertoire breadth is thought to influence brain size: performing more kinds of tasks requires greater processing power (Benson-Amram et al., 2016). A specialist worker of a polymorphic neoattine species would be relatively free of the constraints of maintaining a generalist repertoire and could evolve to prioritize neural capabilities specified by its task set. Size-differentiated workers display disproportionate scaling in morphology and physiology related to social roles that affect task efficiency (Wilson, 1980b). Selection should also be evident in brain structure in both attine clades. In sum, worker morphology, behavior, and brain size and structure are predicted to be integrated.

## Societies, Brains, and Genomes

Ecological niche differentiation, and thus variability in cognitive needs across attine species and among neoattine worker subcastes, is remarkable. In some socially complex species, brain size (Seid et al., 2011), investment in vision-related compartments (Arganda et al., 2020), and microprocessing circuitry (Groh et al., 2014) vary with worker size. Brain volume decreases and antennal lobe volume increases with social group size in monomorphic species, suggesting decreased selective pressures on brain size coupled with a need for increased olfactory social discrimination (Riveros et al., 2012). Larger *Atta* workers have an antennal lobe macroglomerulus, absent in smaller workers, that likely functions in trail following (Kleineidam et al., 2005). Increased volume in visual processing regions in *A. cephalotes* majors allows greater visual acuity and processing in workers active in light and engaging in close-range defense (Arganda et al., 2020). Neuroanatomical and neurochemical variation (Smith et al., 2013) should integrate with brain gene expression to control behavior (Li et al., 2014; Qiu et al., 2018), enabling neural requirements of specific roles to be met.

Genetic analyses offer mechanistic and evolutionary insight into agriculturally adapted brains. Gene expression regulating attine ant neural phenotypes and behavior (Castillo and Pietrantonio, 2013; Koch et al., 2013) may be influenced by epigenetics, RNA editing, and copy number, as in related systems (Chittka et al., 2012; Scholes et al., 2013; Feldmeyer et al., 2014; Li et al., 2014). Developmental switches mediating size-related differentiation (Rajakumar et al., 2012, 2018) and differentially expressed brain gene modules related to caste determination (Qiu et al., 2018) appear conserved, although some worker-biased genes are more evolutionarily novel (Feldmeyer et al., 2014; Mikheyev and Linksvayer, 2015; Schrader et al., 2017). Deep brain homologies in eusocial insects (Tomer et al., 2010; Shpigler et al., 2017; Trible et al., 2017) provide broadly translatable insights into adaptive brain evolution and development, and their genomic basis.

## Future Research

The ability to identify mechanisms of response to cognitive challenges within phylogenetic context facilitates understanding brain evolution in light of socioecological selective forces. This allows the relative importance of task repertoire breadth and social structure to be examined. Studies that assess the same properties of learning (speed and memory, e.g.,) but consider species-specificity in behavior across size-variable workers in paleo-and neoattine ants can elucidate effects of social complexity on brain evolution. Comparative studies of neuroanatomical scaling and genomics enable variation in task diversity and sensory environments to be mapped onto fungus-growing ant phylogeny to reveal evolutionary patterns. Gene functions influencing neuroanatomy and behavior can reveal the relative importance of metabolism, neurotransmission, growth factors, and other pathways in the evolution of division of labor. The contrast between simple societies of monomorphic fungus-growing ants and complex colonies of leafcutting ants provides opportunities to examine genomic evolution in the brain. With increasingly precise genetic tools available for ant research, components of neural and anatomical phenotypes may be separated and linked to developmental origins. Ultimately, functional manipulations and genomic data will enable the identification of neurogenetic traits associated with cognitive evolution.

## Author Contributions

IM and JT drafted and edited the manuscript. JT secured funding. Both authors contributed to the article and approved the submitted version.

## Conflict of Interest

The authors declare that the research was conducted in the absence of any commercial or financial relationships that could be construed as a potential conflict of interest.
